# A Review of Management of *Clostridium difficile* Infection: Primary and Recurrence

**DOI:** 10.3390/antibiotics4040411

**Published:** 2015-09-24

**Authors:** Yasmeen Vincent, Arif Manji, Kathleen Gregory-Miller, Christine Lee

**Affiliations:** 1 St. Joseph’s Healthcare Hamilton, 50 Charlton Ave. East, Hamilton, ON L8N 4A6, Canada; E-Mails: yasmeenvincent@yahoo.ca (Y.V.); manjia@stjosham.on.ca (A.M.); kathleen.gregory@medportal.ca (K.G.-M.); 2 Hamilton Regional Laboratory Medicine Program, Department of Pathology and Molecular Medicine, McMaster University, 1280 Main St. West, Hamilton, ON L8S 4L8, Canada

**Keywords:** recurrence, *Clostridium difficile* infection, fecal microbiota transplantation (FMT), probiotics, immunotherapy, antibiotics

## Abstract

*Clostridium difficile* infection (CDI) is a potentially fatal illness, especially in the elderly and hospitalized individuals. The recurrence and rates of CDI are increasing. In addition, some cases of CDI are refractory to the currently available antibiotics. The search for improved modalities for the management of primary and recurrent CDI is underway. This review discusses the current antibiotics, fecal microbiota transplantation (FMT) and other options such as immunotherapy and administration of non-toxigenic *Clostridium difficile* (CD) for the management of both primary and recurrent CDI.

## 1. Introduction

In a review of the Unites States (US) hospital discharges from 1996 to 2003, there was an increase in *Clostridium difficile-*associated disease from 31/100,000 population in 1996 to 61/100,000 in 2003 [1]. Based on data collected in 2011, Lessa *et al.* reported, in February 2015, that in the United States (US), there are an estimated 453,000 new cases and 83,000 first recurrences of *Clostridium difficile* infection (CDI) [2]. In a 2012 report by Lucado *et al.*, there were 336,600 CDI-related hospital stays in the US, which was 0.9 percent of all hospital stays [3]. Approximately 110,600 cases had CDI as the main cause of hospitalization [3]. In the US and in Canada, the percentage of CDI patients progressing to ICU admissions, colectomy or death ranges from 1% to 5% [4,5,6]. According to the study published in 2014, the estimated attributable costs and length of hospital stays for CDI in the US were as follows: $6774 to $10,212; 5–13.6 days for CDI requiring admission; $2992–$29,000; 2.7–21.3 days for hospital-associated CDI; $2454–$12,850; 2.8–17.9 days where no categorization was made [7]. The costs associated recurrent CDI may significantly exceed that of primary CDI as a single case of recurrent CDI costs $13,655 to $18,067 compared to that of a case of primary CDI at $2871 to $4746 [8].

## 2. Recurrent *Clostridium difficile* Infection

CDI can result in recurrence, either due to relapse or reinfection. An infection due to the same strain of CD which caused the first episode is a relapse, while an infection with a different strain of the organism from the first episode is a reinfection. Relapse usually occurs in a mean of 14.5 days whereas reinfections take a mean of 42.5 days [8,9,10]. Patients who have had a recurrence are at an increased risk of developing subsequent recurrences, which can last months to years. It is postulated that CDIs recur due to the inability of treatment regimens to clear CD spores. Spores can survive in acidic and in antimicrobial environments and within the colonic diverticula [11,12,13,14]. Recurrence occurs when spores germinate and convert to a vegetative form. In healthy adults, the presence of protective colonic microbiota renders CD colonization resistance. A significant disruption of the gut microbiota can allow opportunistic organisms, such as CD, to colonize the colon and cause disease.

### Risk Factors for Recurrent CDI

A three-year study of consecutive cases of new onset CDI at a large referral medical center between 2004 and 2006 revealed 57% of patients who received non-CDI antimicrobials during CDI therapy or within 30 days following the last dose of antibiotic had developed recurrent CDI. From the findings in this study, researchers suggested that antimicrobial exposure of any duration following CDI carries a significant risk of recurrent CDI and therefore unnecessary antimicrobials should be avoided in patients who experienced a recent episode CDI [15]. An additional study conducted between 2003 and 2004 in Quebec, Canada, showed an increasing incidence of recurrence of CDI within 60 days from initial diagnosis when elderly patients were treated with metronidazole alone [16]. McFarland *et al.* reported a subsequent recurrence rate of 44.8% in patients with a history of recurrent CDI treated with standard antibiotics (vancomycin and/or metronidazole) with a median time for recurrence or relapse of eight days [11].

Previous publications identified the risk factors for recurrent CDI as age greater than 65 years, presence of comorbidities such as diabetes and sepsis, stroke, history of previous episodes of recurrence, longer duration of CDI episodes, use of fluoroquinolone, use of clindamycin, antacid or IV glycopeptides, and patients on long-term dialysis. Inability to produce adequate levels of antitoxin A IgG has also been identified as a risk factor [11,16,17,18,19,20]. A more recent retrospective cohort study of 4200 patients with CDI and of these 425 had at least one recurrent CDI. The risk factors for recurrent CDI which were identified on multivariate analyses were high-risk antimicrobial therapy especially fluoroquinolones or intravenous vancomycin following initial CDI treatment; community-onset healthcare-associated CDI and gastric suppression; two or more hospitalization within prior 60 days and age [21].

## 3. Treatment Options for CDI

Treatment of recurrent CDI remains a challenge. There is no single treatment that is effective for all CDI cases. This has led researchers to search for alternate treatment options [11]. Metronidazole and vancomycin remain the standard treatment for initial and first recurrent episode of CDI. For second recurrences pulsed or tapering doses of vancomycin is recommended. For recurrence beyond the second episode, a number of other treatment strategies including fecal microbiota transplantation (FMT), bacteriotherapy, probiotics, immunotherapy, and newer drugs have been tried [22,23].

### 3.1. Antibiotics

#### 3.1.1. Metronidazole

Oral metronidazole is recommended as the first line therapy for uncomplicated, initial or 1st recurrent CDI. However, a ten-year study in Canada between 1991 and 2002 revealed a metronidazole failure rate of 9.6%. This trend increased to 25.7% between 2003 and 2004. These researchers also noted an increase in the frequency of post metronidazole therapy recurrence as compared to that seen between 1991 and 2002 [16]. This increase was possibly due to the ongoing outbreak with the hypervirulent fluoroquinolone resistant strain of CD the B1/NAP1/027 strain, which could have developed increased resistance to metronidazole due to an increase in physician request for toxin assay or because patients were given repeat treatment a second time once diarrhea recurred which caused an ascertainment bias [16]. Musher *et al.*, in 2005, reported a metronidazole failure rate of 22% in Houston [24]. Recently, Johnson *et al.* reported the findings of two large multinational randomized controlled trials (RCT), comparing vancomycin, metronidazole, and tolevamer for the treatment of CDI. Findings from these studies revealed that metronidazole is inferior to vancomycin based on clinical cure adjusted for other relevant factors (including disease severity). This was most evident in patients with severe disease although the difference was not statistically significant (*p* = 0.059) [25]. They found no difference in the recurrence rates between metronidazole and vancomycin during this study for initial CDI. Interestingly they did demonstrate a trend although not statistically significant for vancomycin to cause less recurrences compared to metronidazole for recurrent CDI (*p* = 0.08) [25]. A systematic review to evaluate the rates of treatment failure and recurrence of CDI following vancomycin or metronidazole over a 10-year period showed that the treatment failure were 22.4%; 14.2% and recurrence of 27.1% and 24.0% after treatment with metronidazole and vancomcyin, respectively [26].

It was previously suggested that intravenous metronidazole may be administered along with intraluminal vancomycin in patients with severe disease when oral administration is not possible [27]. However, findings from a more recent non-randomized study revealed a lower cure rate and a higher incidence of mortality with intravenous metronidazole as compared to oral metronidazole and oral vancomycin in mild CDI; therefore, the recommendation is to avoid intravenous metronidazole alone for the management of CDI and whenever oral therapy is possible [28].

#### 3.1.2. Glycopeptides

##### Vancomycin

Vancomycin has shown good activity against CD both *in vivo* and *in vitro* [26]. Zar *et al.* compared metronidazole and vancomycin for the management of CDI in 172 patients stratified by disease severity between 1994 and 2002 [29]. Although they did not find any difference in efficacy between the two drugs for the management of mild CDI they found that vancomycin was superior to metronidazole in the management of severe CDI [29]. Most patients who develop a second and subsequent recurrence of CDI are prescribed vancomycin [29,30,31]. McFarland *et al.* studied a cohort of 163 patients with recurrent CDI [11]. They reported a significant difference in the rate of recurrent CDI depending on the vancomycin regimen administered. Three regimens of vancomycin were provided: varying doses of vancomycin given over one to two weeks, tapered doses of vancomycin and pulsed doses of vancomycin. The findings from this study indicate that the best response is achieved with pulsed and/or tapered doses of vancomycin [11]. Pulsed and or tapering dosing may be more effective due to the germination of spores into the vegetative form of the organism, which are then killed by the higher pulsed dose. The tapering dose may also provide an opportunity for the restoration of healthy microbiota of the gut. Tedesco *et al.*, in 1985, reported an absence of relapse during a 2- to 12-month follow-up of 22 patients with relapsing CDI who were treated with either tapered or pulsed doses of vancomycin over a 21-day period [32].

##### Teicoplanin

Wenisch *et al.*, in 1995, reported that treatment of CDI with teicoplanin was as effective as metronidazole and showed lower recurrence rates compared to other antimicrobials. They suggested a dose of 200 mg/day administered orally [27]. The limitations of use of teicoplanin for CDI are the high cost and insufficient clinical data for its use in management of CDI.

#### 3.1.3. Rifamycins

Rifaximin is a derivative of rifamycin and its mechanism of action is through the inhibition of bacterial transcription and protein synthesis. In small trials, rifaximin at doses of 100 to 200 mg, twice daily for two weeks in patients previously treated with oral vancomycin, has shown promising results in the prevention and treatment of recurrent CDI [33,34,35]. However, larger studies are required to corroborate these findings.

#### 3.1.4. Fidaxomicin

Fidoxamicin is an oral, macrocyclic, narrow spectrum antibiotic with minimal systemic absorption, high fecal concentrations [34,35,36,37] and bactericidal activity [38]. *In vitro* it has demonstrated higher activity against *C. difficile* strains including NAP1/BI/027. It also been shown (both *in vitro* and *in vivo*) that it has negligible activity against bowel normal flora which protect against multiplication of CD) [39]. A large RCT has shown that the rate of recurrence is lower than oral vancomycin for non-NAP 1 strain-related initial or 1^st^ recurrent cases of CDI [35,36,37,40]. Crooks *et al.* reported the findings of two prospective, more recent, multicenter, double-blind, randomized, parallel group studies comparing fidaxomicin to vancomycin in managing cases of CDI [39]. A total of 1164 patients with active CDI were randomized to receive blinded oral therapy as either 200 mg of fidaxomicin every 12 h with intervening matching doses of placebo or 125 mg of vancomycin every 6 h for a total of 10 days. Data from the two studies were analyzed by three methods, intention-to-treat (ITT), modified intention to treat (mITT) and per protocol analyses [39]. ITT analyses revealed that fidaxomicin reduced persistent diarrhea, recurrence or death by 40% (95% confidence interval (CI), 26%–51%; *p* < 0.0001) through day 40. On the other hand, mITT and per-protocol analyses of clinical failure/cure (assessed at day 12) suggested statistically insignificant (*p* = 0.40 and *p* = 0.33, respectively) early benefits associated with fidaxomicin *vs.* vancomycin. The study showed that CDI in the previous three months was the only strong risk factor for recurrence (*p* < 0.01) between days 13 and 40. In this study, fidoxamicin was non-inferior to vancomycin for primary episode of CDI but there were fewer recurrences with fidoxamicin compared to vancomycin [39]. A single-center, retrospective case series consisting of 22 cancer patients responded to fidaxomicin after failing standard antibiotic therapy for primary episode of CDI [41].

### 3.2. Immune Therapy

Findings from a study in Quebec showed that patients with more than 30 days of hospitalization had reduced risk of developing recurrence of CDI. Researchers suggested that this could be due to a development of immunity due to repeated exposures to the organism or due to a selection process [16]. Immunotherapy has been tried as an adjunct to standard treatment for recurrent CDIs.

#### 3.2.1. Active Immunization

Evidence from a small case report of three patients with recurrent CDI has shown that vaccination against toxin A and B may be another option for the treatment of recurrent CDI. These patients were administered CD toxoid A and B during treatment with oral vancomycin [42].

#### 3.2.2. Passive Immunization

Intravenous immune globulin against toxin A and toxin B of CD has been used to treat recurrent CDIs in conjunction with metronidazole or vancomycin. Findings from a large RCT revealed that patients with CDI who were given one infusion of antibodies against both toxins A and B, each at 10 mg/kg body weight along with either metronidazole or vancomycin had a 72% lower recurrence rate as compared to patients who were given a placebo [43].

### 3.3. Microbial Therapy

#### 3.3.1. Probiotics

Probiotics are live microbial organisms that are bacterial-based, such as *Bifidobacteria* spp. and *Lactobacillus* spp., or yeasts such as *Saccharomyces boulardii* [44]. They are attributed to be able to re-establish normal gut flora. There are insufficient data to support the use of probiotics for the management of recurrent CDI because there is a lack of properly conducted clinical trials. Conclusions from a Cochrane study indicate that there is insufficient evidence to recommend the use of probiotics as an adjunct to antimicrobial therapy for the prevention and treatment of recurrent CDI [45]. A few studies have shown that *Saccharomyces boulardii* may be useful in preventing the recurrence of CDIs when used along with other modalities of treatment mainly antimicrobials like vancomycin [18,46] but these findings need to be confirmed by larger RCTs. Some researchers have recommended that immunocompromised patients should not use probiotics due to the risk of developing fungemia [47,48].

#### 3.3.2. Bacteriotherapy

Bacteriotherapy is the introduction of normal gut bacterial flora harvested from the stool of healthy donor or from cultures into the colon of a patient to restore intestinal homeostasis.

##### Fecal Microbiota Transplantation

FMT was first described in Chinese literature in the 4th century [49]. Schwan *et al.*, in 1983, described for the first time the management of relapsing *C. difficile* enterocolitis using FMT by enema [50].

Healthy intestinal microbiota suppress the growth of pathogenic organisms. CDI occurs when there is loss of healthy microbiota, commonly due to antibiotic therapy, which allows propagation of CD. FMT replaces healthy microbiome and provides an opportunity for the intestines to re-establish healthy microbiome [51,52,53]. It can be used in the cure of CDI however most interest is in the prevention of recurrent CDI.

FMT has shown to be safe, inexpensive and effective [54]. Van Nood *et al.* conducted the first RCT on FMT and found FMT was superior to vancomycin for disease resolution [55]. Various organizations have provided recommendations for using FMT in the management of recurrent CDI. The 2010 Society for Healthcare Epidemiology of America (SHEA), Infectious Diseases Society of America (IDSA) CDI clinical practice guidelines, and the 2013 American College of Gastroenterology (ACG) 2013 CDI guidelines recommend the use of FMT for the treatment of third recurrence, following a tapered and/or pulsed vancomycin treatment for a second recurrence that fails. The ACG also recommends the use of FMT for managing multiple recurrent CDIs [23,56]. The cure rate of multiple recurrent CDIs following FMT has been described at 90%. From available literature, this appears to be the most promising method for the management of recurrent CDI. Kassam *et al.* conducted a meta- analysis of 16 case series with a total of 526 CDI patients who did not respond to antibiotics and were treated with FMT. The cure rates in these series ranged between 69% and 100% and there was clinical resolution in 88% of patients [57].

The major concern with regard to FMT is the possible transmission of infectious agents from donor stool to patient. In order to minimize this risk, a rigorous screening is conducted by questionnaire, blood and stool tests for every potential donor. An additional concern is the possibility of transmission of autoimmune diseases. These concerns highlight the need for long term follow up of recipients of FMT [57,58].

FMT can be administered either through the upper or lower gastrointestinal (GI) route. Administration via a nasogastric tube is the most commonly used upper GI method and colonoscopy or enema are the methods used to deliver FMT via the lower GI tract. Other methods include naso-enteric, gastro-duodenoscopy, sigmoidoscopy and gastroscopy [59,60].

Youngster *et al.* conducted a RCT of ten patients comparing the nasogastric *vs.* the colonoscopy route of FMT administration [61]. Patients treated via colonoscopy achieved an 80% clinical cure rate, compared to the 60% cure rate for those treated via the nasogastric route. No recurrence or relapse was observed in clinically cured patients in either arm in the eight week follow up period [61]. Gastrointestinal perforation is a risk in both these methods. As compared to the above two methods, retention enema does not deliver stool to all areas of the colon but it is less invasive, there is no risk of intestinal perforation, it is cheaper, easier to perform and can be easily administered without much expertise. Clinical cure rate has been found to be higher as compared to delivery by nasogastric tube or colonoscopy [53,62,63]. However, FMT administration by enema may not be effective in patients who cannot retain the infusate due to poor sphincter tone [63]. Silverman *et al.* have suggested that retention enema can be successfully administered by the patient or a family member, although another group of researchers have reported that some patients find handling stool unacceptable [64,65].

Wettstein *et al.*, in 2007, conducted a retrospective study of 16 patients with relapsing CDI managed with colonoscopy delivered FMT. There was a 93.75% cure rate in these patients in whom antimicrobial therapy had failed to resolve the infection [66]. Guo *et al.* reviewed seven case series with a total of 124 patients with recurrent CDI conducted between 2000 and 2001. Results from these studies revealed that FMT was safe and effective and 83% of patients experienced immediate symptom relief [67]. In 2012, Kassam *et al.* reported a case series of 27 patients with refractory or recurrent CDI treated with fecal transplantation via retention enema. The clinical cure rate reported in this series was 93% with no relapse in the follow up period of 427.3 days. Treatment failure occurred in two of the 27 patients [63].

FMT has traditionally been administered as a fresh preparation. However problems with fresh stool preparations include difficulty in preparation due to time constrains regarding collection and administration. Jiang *et al.* studied the efficacy of fresh, frozen and lyophilized FMT preparations. They found no difference in the donor stool quality between fresh and frozen preparations and lyophilized FMT was as effective as fresh stool in treating recurrent CDI [68].

#### 3.3.3. Non Toxigenic Clostridium difficile Spores

The administration of non-toxigenic strain of CD spores has been studied as a method for the prevention of recurrent CDI. Gerding *et al.* studied the safety and efficacy of *C. difficile* M3 (VP20621; NTCD-M3), a non-toxigenic strain of CD administered as an oral liquid formulation. This was a phase 2, randomized double blind placebo controlled dose ranging clinical trial conducted between 2011 and 2013 with a total of 173 patients. These patients had experienced a first episode or first recurrence of CDI and had successfully been treated with metronidazole, vancomycin or both. Patients were studied at 44 centers across Europe, the United States, and Canada. The conclusions from this study were that NTCD-M3 was safe, well tolerated, colonized the gastrointestinal tract and significantly reduced the recurrence of CDI to 11% from 30% in the placebo arm (*p* = 0.006) [69].

## 4. FMT Clinical Trials

The search for an effective, safe and aesthetically acceptable treatment options for recurrent CDI is ongoing. Among the treatment modalities examined thus far, fecal microbiota transplantation has shown to be most effective in preventing CDI recurrences and appears to be safe [70]. There are a number of ongoing FMT clinical trials for the management of recurrent CDI.

A group of researchers at the University of Alberta are comparing the delivery of FMT by capsule *vs.* colonoscopy in a prospective multicenter RCT; with the anticipated completion in August 2016 [71]. A phase 2, prospective trial to study the cure rate, safety, relapse and failure rates, and a 10-year follow-up for long-term effects of FMT administered via enema for the management of recurrent CDI is being conducted at McMaster University, Canada. [72]. The recurrence rate following FMT therapy by colonoscopy, sigmoidoscopy or retention enema in patients of recurrent CDI is being studied by a group of researchers at Englewood Hospital and Medical Center, USA. This study is expected to be completed in December 2015 [73].

## 5. Conclusions

CDI is increasing in incidence. Severity and recurrence of infection is a major challenge. The use of standard antibiotic therapy is ineffective in the prevention and management of recurrent CDI. FMT has shown to be the most effective and safe option in the management of recurrent CDI. A multitude of studies are underway to discover the optimal FMT.
